# Authors reply in response to a letter on: “Diagnostic yield, safety and therapeutic consequences of myocardial biopsy in clinically suspected fulminant myocarditis unweanable from mechanical circulatory support”

**DOI:** 10.1186/s13613-023-01237-3

**Published:** 2024-01-06

**Authors:** Marc Pineton de Chambrun, Yann Marquet, Mathieu Kerneis, Matthieu Schmidt, Charles-Edouard Luyt, Alain Combes, Guillaume Hekimian

**Affiliations:** 1 Service de Médecine Intensive-Réanimation, Sorbonne Université, Assistance Publique-Hôpitaux de Paris (AP-HP), Hôpital La Pitié–Salpêtrière, 47–83, Boulevard de l’Hôpital, 75651 Paris Cedex, France; 2 Institut de Cardiométabolisme et Nutrition (ICAN), Sorbonne Université, INSERM, UMRS_1166-ICAN, F-75013 Paris, France; 3 Service de Médecine Interne 2, Centre de Référence National Lupus Systémique, Syndrome des Anticorps Anti-Phospholipides et Autres Maladies Auto-Immunes Systémiques Rares, Sorbonne Université, AP-HP, Hôpital La Pitié–Salpêtrière, Institut E3M, Paris, France; 4 Sorbonne Université, Inserm, Centre d’Immunologie et des Maladies Infectieuses (CIMI-Paris), Paris, France; 5 ACTION Study Group, Département de Cardiologie, Sorbonne Université, AP-HP, Hôpital La Pitié-Salpêtrière, Paris, France

We read with interest the letter by Giordani et al*.* commenting on our recent report [1]. In this article, we address the low diagnostic value, the few therapeutic consequences, and the high complication rate of endomyocardial biopsy (EMB) and surgical myocardial biopsy in patients with fulminant clinically suspected myocarditis (FCSM) on temporary mechanical circulatory support (t-MCS).

First, our inclusion criteria were designed to stick with the “real-life” use of EMB. As guidelines are based on a low level of evidence, EMB is not routinely performed in FCSM worldwide, especially since many patients spontaneously and quickly recover. Our diagnostic algorithm advocates EMB only in patients for whom a diagnosis was not proven by less-invasive techniques or did not rapidly recover. In our series, the median [IQR25-75] time from hospital to intensive care unit (ICU) admission was 1 [0–4] days with a time from ICU to biopsy of 6 [3–11] days. This delay is consistent with the usual time needed for many patients to spontaneously recover and get weaned from t-MCS.

Second, a recent international retrospective study seriously challenged the usefulness of EMB. Over 419 patients (77% under t-MCS) with FCSM, the unadjusted 1-year transplant or ventricle assist device-free survival was identical between patients undergoing early EMB (≤ 48 h after ICU admission, *n* = 103, 70%) and those without EMB (*n* = 236, 69%) [2]. This striking finding challenges the therapeutic impact of EMB. Only patients with late EMB (≥ 48 h after ICU admission, 49%) had a poorer outcome, which could be close to our population, namely those undergoing EMB because of no early recovering cardiac function. Unfortunately, the propensity scores matched-comparison in this study focused only on patients who had EMB, therefore excluding patients with no EMB-proven myocarditis (but proven by other tools like MRI and non-invasive viral samples, etc.). This choice has precluded the relevant comparison of the outcomes between patients without EMB and those who had early or late EMB. This comparison is eagerly expected to better guide our clinical practices, especially on patients with t-MCS.

Third, achieving a diagnosis of certainty is useless if no beneficial therapeutic interventions arise from it, while most of the non-ischemic acute left ventricular dysfunctions have no specific treatment. Excluding myocardial infarction is mandatory as it could drastically change patients’ management. While viral infection can hardly be excluded even with the most recent metagenomic next-generation techniques, viral infection frequently causing myocarditis can be diagnosed with a simple nasopharyngeal viral multiplex RT-PCR. To date, no viral treatment has been shown effective in treating viruses-induced FCSM. Elevated blood eosinophil count is present in most eosinophilic myocarditis and this information is frequently sufficient to start a promptly efficient corticosteroid treatment [3]. “Autoimmune virus-negative myocarditis” is not a consensual clinical entity. Systemic autoimmune diseases with cardiac involvement—systemic lupus erythematosus, vasculitis, inflammatory idiopathic myositis—are diagnosed and treated without EMB (clinical examination, immunological laboratory work-up, etc.). Last, there is no evidence of immunosuppressant efficacy in non-viral, non-autoimmune disease-associated lymphocytic myocarditis. The case scenario of giant-cell myocarditis requiring t-MCS is archetypical. Beyond the scarcity of this etiology (i.e. less than 5% of FCSM [2]), EMB is mandatory to confirm the diagnosis and initiate a specific treatment whose effectiveness has been seriously challenged in patients on t-MCS [4].

Fourth, we agree that a few patients from our study might have benefited from EMB and we do not advocate against EMB in every patient. A wise approach should carefully weight the benefits and the risks on a case-to-case basis as we always do with any invasive technique in the ICU.

Fifth, we used the internationally validated histopathological criteria for the diagnosis of myocarditis integrated in the Bonaca criteria for myocarditis [5]. These criteria are very innovative and have a high real-life applicability, as they recognize definite myocarditis even without EMB.

Finally, we report a higher rate of complications of EMB as our patients were under t-MCS. T-MCS requires anticoagulation and is responsible for device-related thrombocytopenia and/or coagulation disorders. The previously reported rates of EMB complications come from studies with retrospective design, in which adverse events are frequently underreported. Furthermore, these data arise from high-volume EMB centers and do not apply to low-volume centers. The current evidence is not sufficient to determine the true frequency of complications following EMB and this should be considered when appraising the benefit–risk ratio of EMB in critically-ill patients.

In conclusion, given the few specific treatments for fulminant myocarditis, the diagnostic and therapeutic contributions of EMB in FCSM on t-MCS appear low. Guidelines supporting the use of EMB in this setting still rely on expert opinion supported by a low level of scientific evidence and based on studies that frequently excluded patients with no EMB-proven myocarditis. To date, it is still uncertain if EMB can change FCSM outcomes. Randomized clinical trials are now needed to investigate this important point for clinical practices.

## Data Availability

Not applicable.

## Abbreviations

FCSM: Fulminant clinically suspected myocarditis
EMB: Endomyocardial biopsy
GCM: Giant cell myocarditis
ICU: Intensive care unit
IQR: Interquartile range
t-MCS: Temporary mechanical circulatory support
PCR: Polymerase chain reaction

## References

[1] Marquet Y, Hékimian G, Lebreton G, Kerneis M, Rouvier P, Bay P (2023). Diagnostic yield, safety and therapeutic consequences of myocardial biopsy in clinically suspected fulminant myocarditis unweanable from mechanical circulatory support. Ann Intensive Care.

[2] Huang F, Ammirati E, Ponnaiah M, Montero S, Raimbault V, Abrams D (2023). Fulminant myocarditis proven by early biopsy and outcomes. Eur Heart J.

[3] Brambatti M, Matassini MV, Adler ED, Klingel K, Camici PG, Ammirati E (2017). Eosinophilic myocarditis: characteristics, treatment, and outcomes. J Am Coll Cardiol.

[4] Montero S, Aissaoui N, Tadié J-M, Bizouarn P, Scherrer V, Persichini R (2018). Fulminant giant-cell myocarditis on mechanical circulatory support: Management and outcomes of a French multicentre cohort. Int J Cardiol.

[5] Bonaca MP, Olenchock BA, Salem J-E, Wiviott SD, Ederhy S, Cohen A (2019). Myocarditis in the setting of cancer therapeutics: proposed case definitions for emerging clinical syndromes in cardio-oncology. Circulation.

